# Severe Bradycardia Increases the Incidence and Severity of Torsade de Pointes Arrhythmias by Augmenting Preexistent Spatial Dispersion of Repolarization in the CAVB Dog Model

**DOI:** 10.3389/fphys.2021.642083

**Published:** 2021-04-26

**Authors:** Valerie Y. H. van Weperen, Albert Dunnink, Alexandre Bossu, Jet D. M. Beekman, Veronique M. F. Meijborg, Jacques M. T. de Bakker, Ruben Coronel, Rosanne Varkevisser, Marcel A. G. van der Heyden, Marc A. Vos

**Affiliations:** ^1^Department of Medical Physiology, Universitair Medisch Centrum Utrecht, Utrecht, Netherlands; ^2^Department of Clinical and Experimental Cardiology, Amsterdam UMC Locatie AMC, Amsterdam, Netherlands

**Keywords:** CAVB dog model, sudden cardiac death, temporal dispersion of repolarization, spatial dispersion of repolarization, arrhythmogenesis, severe bradycardia, Torsade de Pointes

## Abstract

**Introduction:**

Torsade de pointes arrhythmias (TdP) in the chronic atrioventricular block (CAVB) dog model result from proarrhythmic factors, which trigger TdP and/or reinforce the arrhythmic substrate. This study investigated electrophysiological and arrhythmogenic consequences of severe bradycardia for TdP.

**Methods:**

Dofetilide (25 μg/kg per 5 min) was administered to eight anesthetized, idioventricular rhythm (IVR) remodeled CAVB dogs in two serial experiments: once under 60 beats per minute (bpm), right ventricular apex paced (RVA60) conditions, once under more bradycardic IVR conditions. Recordings included surface electrocardiogram and short-term variability (STV) of repolarization from endocardial unipolar electrograms. TdP inducibility (three or more episodes within 10 min after start of dofetilide) and arrhythmic activity scores (AS) were established. Mapping experiments in 10 additional dogs determined the effect of lowering rate on STV and spatial dispersion of repolarization (SDR) in baseline.

**Results:**

IVR-tested animals had longer baseline RR-interval (1,403 ± 271 ms) and repolarization intervals than RVA60 animals. Dofetilide increased STV similarly under both rhythm strategies. Nevertheless, TdP inducibility and AS were higher under IVR conditions (6/8 and 37 ± 27 vs. 1/8 and 8 ± 12 in RVA60, respectively, both *p <* 0.05). Mapping: Pacing from high (128 ± 10 bpm) to middle (88 ± 10 bpm) to experimental rate (61 ± 3 bpm) increased all electrophysiological parameters, including interventricular dispersion, due to steeper left ventricular restitution curves, and intraventricular SDR: maximal cubic dispersion from 60 ± 14 (high) to 69 ± 17 (middle) to 84 ± 22 ms (*p* < 0.05 vs. high and middle rate).

**Conclusion:**

In CAVB dogs, severe bradycardia increases the probability and severity of arrhythmic events by heterogeneously causing electrophysiological instability, which is mainly reflected in an increased spatial, and to a lesser extent temporal, dispersion of repolarization.

## Introduction

Ventricular arrhythmias comprise a multitude of life-threatening conditions that often result in sudden cardiac death (SCD) (Morin and Homoud, 2017). Much research is therefore dedicated to deciphering the complex processes that collectively result in the generation and perpetuation of ventricular arrhythmias and the development of possible therapies to prevent such events.

The employment of the chronic atrioventricular block (CAVB) dog model has resulted in great advances in the research field of ventricular arrhythmias. The reproducibility of torsade de pointes arrhythmias (TdP) in this model results from complex ventricular adaptation processes, initiated by ablation of the proximal His bundle. The subsequent chronic drop in heart rate and thus cardiac output, combined with an altered ventricular activation pattern, initiate contractile, structural, and electrical remodeling processes. Cumulatively, these adaptations reestablish an adequate cardiac output but simultaneously, and adversely, also increase susceptibility for TdP (Oros et al., 2008; Dunnink et al., 2012).

Arrhythmogenesis in the CAVB dog is stimulated by multiple factors that promote the development of triggered activity and/or modulate the arrhythmogenic substrate. Even though both mechanisms are essential for the generation of TdP, they serve different purposes herein. The trigger initiates ectopic activity and originates from early or late after-depolarizations at the cellular level. Normally, a redundancy in the repolarization machinery, known as repolarization reserve, hinders the development of such erroneous after-depolarizations as it allows myocytes to compensate for repolarization impeding or challenging circumstances (Roden, 1998; Varró and Baczkó, 2011). However, in the CAVB dog model, electrical remodeling (Volders et al., 1999; Takahara et al., 2011), which includes down-regulation of repolarization currents *I*_Kr_ and *I*_Ks_, causes this reserve to become chronically impaired. As such, electrical instability induced by additional debilitating factors, such as infusion of a proarrhythmic drug, is inadequately compensated for, resulting in the appearance of triggered activity and TdP (Kozhevnikov et al., 2002; Schreiner et al., 2004; Oros et al., 2008; Dunnink et al., 2012). Short-term variability (STV) of repolarization reflects the condition of the repolarization reserve; the more it is diminished, the higher the STV (Thomsen et al., 2004; Antoons et al., 2015). In addition, progression of these ectopic beats to more severe arrhythmic events also relies on the presence of sufficient spatial dispersion of repolarization (SDR) (Dunnink et al., 2017). This heterogeneity in repolarization duration has been demonstrated to be involved in the propagation of ectopic events and to be of increasing importance for the evolution of ectopic events to TdP (Dunnink et al., 2017).

Identification of factors that reduce repolarization reserve is of great importance for the complete understanding of ventricular arrhythmias and might have important clinical and experimental implications. In both the clinical and experimental setting, bradycardia has been acknowledged to be one of such repolarization reserve challenging factors. In fact, when Dessertenne first described TdP in 1966 (Dessertenne, 1966), the mentioned episode also developed under bradycardic conditions. Moreover, tachypacing has been established to be an effective arrhythmia suppressor in both the clinical and experimental settings (Wathen et al., 2001; Wijers et al., 2017; Smoczyńska et al., 2020). Despite this clear evidence on the antiarrhythmic properties of increasing heart rate, the exact arrhythmogenic and electrophysiological consequences of severely decreasing heart rate have been much less explored.

This study therefore specifically aimed to *in vivo* quantify and reaffirm the proarrhythmic character of severe bradycardia and to establish its electrophysiological effects on STV and SDR in three dimensions. As such, eight CAVB dogs were serially subjected to a proarrhythmic challenge with the specific *I*_Kr_ blocker dofetilide. One experiment was conducted under idioventricular rhythm (IVR) conditions, anesthesia, and dofetilide inducing further slowing of IVR. In the other experiment, all animals were continuously paced at 60 beats per minute (bpm) from the right ventricular apex (RVA60). Additionally, detailed *in vivo* mapping experiments were conducted under different pacing frequencies to elucidate the effect of pacing frequency on SDR.

## Materials and Methods

All experiments were approved by the Committee for Experiments on Animals of Utrecht University, Netherlands. Animal handling and care wEre in accordance with the European Directive for the Protection of Vertebrate Animals Used for Experimental and Scientific Purposes 2010/63/EU of the European Parliament and Council of September 22, 2010. This study included a total of 18 adult purpose-bred mongrel dogs (15 females, 3 males; average weight 24 ± 3 kg; Marshall, United States).

Animals were housed in conventional dog kennels enriched with wooden bedding and playing tools. If possible, dogs were housed in pairs and let out of the kennel at least once a day to go outside and play. The animals had *ad libitum* access to water and were provided with dog food pellets twice a day. Daily checks on health and comfort were performed; weight was measured once a week.

### Animal Preparation

Premedication consisted of 0.5 mg/kg methadone, 0.5 mg/kg acepromazine, and 0.02 mg/kg atropine [intramuscular (i.m.)]. In addition, prophylactic antibiotic ampicillin (1,000 mg) was given before and after the experiment, and perioperative analgesics consisted of meloxicam (Metacam) (0.2 mg/kg subcutaneously preoperatively) and buprenorphine (0.3 mg i.m., postoperatively). General anesthesia was induced using pentobarbital sodium [25 mg/kg intravenously (i.v.)] and maintained with mechanical ventilation of isoflurane (1.5%) in a mixture of O_2_ and N_2_O (1:2).

A pacemaker screw-in lead was introduced through the jugular vein and positioned in the RVA after which it was connected to an internal pacemaker (Medtronic, Maastricht, Netherlands). Subsequent radiofrequency ablation of the proximal His-bundle induced complete AV block. All animals were allowed a minimum remodeling period of 3 weeks. All animals remodeled under continuous IVR conditions except for three dogs used in the mapping experiments, which remodeled under continuously RVA paced conditions at the lowest captured rate. Experiments included in the serial study were performed 5.6 ± 1.8 weeks after creation of AV block, whereas the mapping experiments were performed 14.1 ± 4.6 weeks after creation of AV block.

Electrophysiological recordings were continuously made during all experiments and consisted of a standard six-lead electrocardiogram (ECG) with four additional precordial leads, right ventricular (RV) monophasic action potential (MAP) catheters (Hugo Sachs Elektronik GmbH, March, Germany; not in mapping experiments), and either a left ventricular (LV) MAP catheter or a duo-decapolar catheter (St. Jude Medical, St. Paul, MN, United States) recording unipolar electrograms (EGMs) from 10 distinct endocardial regions in the LV. The measurements derived from a MAP or an EGM catheter are interchangeable (Oosterhoff et al., 2010; Wijers et al., 2018).

### Experiments

#### Dofetilide Experiments

Eight animals were subjected to a dofetilide challenge twice; in one experiment, animals were in IVR, anesthesia, and dofetilide slowing heart rate, whereas the other experiment was performed under RVA60 conditions.

The dofetilide challenge was performed as previously described by Bossu et al. (2018). In short, 10 min of baseline electrophysiological recordings were followed by administration of dofetilide (0.025 mg/kg per 5 min; i.v.), a specific *I*_Kr_ blocker, to assess inducibility for TdP. Electrophysiological recordings were continued for a minimum of 10 min following start of infusion. In experiments where TdP occurred within 5 min of dofetilide infusion, administration was immediately discontinued. In addition, persistent arrhythmias (>10 s) were manually terminated by electrical cardioversion, applied *via* thoracic patches.

#### Mapping Experiments

*In vivo* mapping experiments were performed in 10 other animals to assess the effects of acute bradycardia on SDR under baseline conditions. In these experiments, 56 needles with each four recording electrodes (interelectrode distance 0.4 mm) were evenly inserted into the LV and RV walls (Dunnink et al., 2017). All animals were continuously paced from the RVA, and SDR was assessed as pacing frequency was decreased from high (128 ± 10 bpm; similar to canine sinus rhythm) to middle (88 ± 10 bpm) to experimental (61 ± 3 bpm) rate.

### Data Analysis

Electrophysiological data obtained from the surface ECG (RR, QRS, and QT intervals) were analyzed using EPTracer software (Cardiotek, Maastricht, Netherlands). All intervals were determined from lead II of the surface ECG and measured manually from five consecutive beats during baseline and either prior to the first ectopic beat or at 5 min following start of dofetilide infusion. QT interval was corrected for heart rate (QTc) using the Van der Water formula (Van de Water et al., 1989). Additionally, the interval between the peak of the T-wave and the end of the T-wave (Tp-e) and the interval between the J-point and the T-peak (JTp) were measured as parameters reflective of early and global SDR, respectively (Opthof et al., 2007; Johannesen et al., 2014).

**FIGURE 1:**
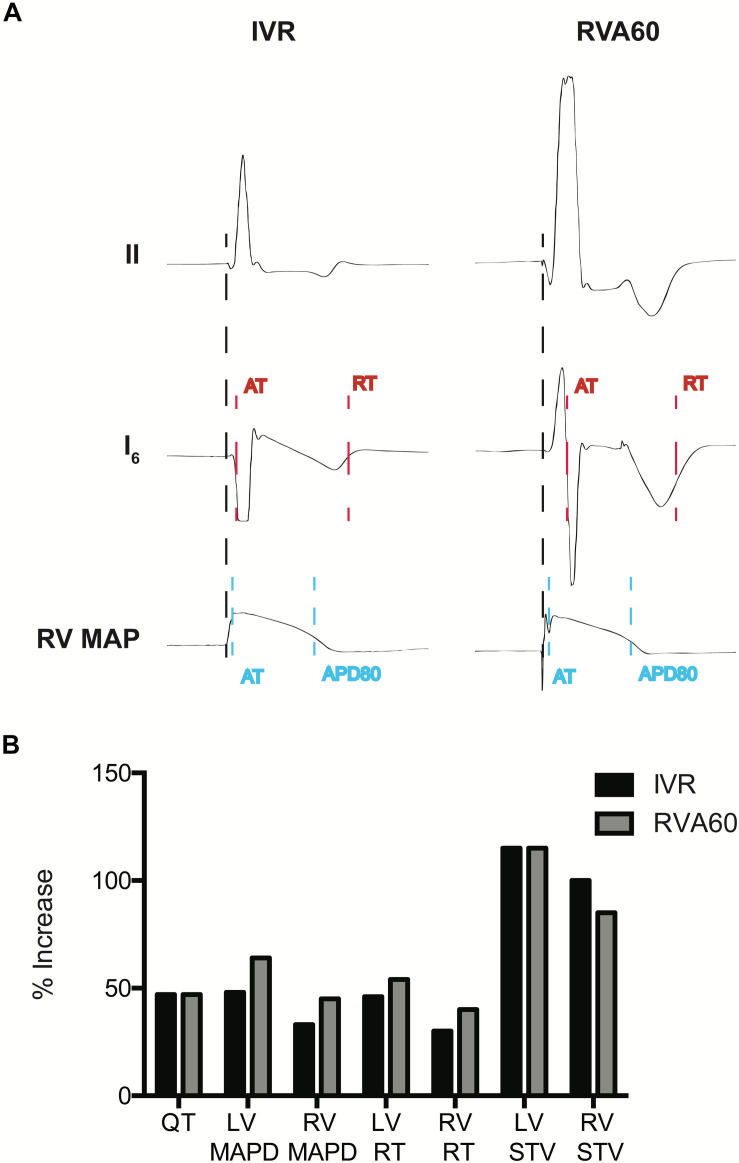
Measurement of electrophysiological parameters and the percentage (%) increases of electrophysiological parameters following infusion of dofetilide in the serially performed IVR and RVA60 experiments. **(A)** Representative beats from lead II, a left ventricular (LV) intraventricular electrogram (EGM) and a right ventricular (RV) monophasic action potential (MAP) in IVR and RVA experiments. Start of activation (black dashed line) was determined at the start of the QRS complex (IVR experiments) or pacing spike (RVA experiments) in lead II. LV activation time (AT) was measured as the time between start of ventricular activation and the steepest downslope of the EGM (red dashed line), whereas LV repolarization time (RT) was measured at the steepest upslope (red dashed line). LV activation recovery interval (ARI) was measured as LV, RT – LV, AT. RV, AT was measured from the start of ventricular activation to the upstroke of the RV MAP (blue dashed line). Monophasic action potential duration (MAPD) was determined at 80% of repolarization (blue dashed line). **(B)** The percentage in electrophysiological parameters representing repolarization time and stability following infusion of dofetilide in the serially performed IVR and RVA60 experiments. IVR, idioventricular rhythm; LV/RV MAPD, left/right ventricular monophasic action potential duration; LV/RV RT, left/right ventricular repolarization time; LV/RV STV, left/right ventricular short-term variability; RVA60, right ventricular apex paced at 60 beats per minute.

Activation time (AT) was manually determined as the time difference between the start of the QRS complex (IVR) or pacing spike (RVA60) and the steepest upstroke of the MAP or the steepest downstroke of the QRS in the EGM (Figure 1A). The MAP durations (MAPDs) were determined at 80% repolarization using a custom-made MATLAB application (MathWorks, Natick, MA, United States) (Figure 1A). The same software was used to measure LV activation recovery intervals (ARIs), which were obtained from a unipolar EGM from the most apical located electrode of the duo-decapolar catheter (Figure 1A). A duo-decapolar catheter was used in eight and six experiments of the serial comparison and mapping experiments, respectively.

STV of repolarization was calculated from 30 consecutive beats using the formula:

$$\text{STV}=\frac{\sum \left|D_{n+1}-\,D_{n}\right|}{30\,x\,\sqrt{2}}$$

with *D* being LV ARI or LV MAPD (LV ARI/MAPD) or RV MAPD (Thomsen et al., 2004). Interventricular dispersion of APD (ΔAPD) was calculated as LV ARI/MAPD – RV MAPD. Repolarization time (RT) was obtained by summation of the AT and MAPD, or ARI. Interventricular difference between AT (ΔAT) and RT (ΔRT) was calculated as LV-AT or -RT – RV-AT or -RT, respectively.

The unipolar EGMs of the mapping experiments were analyzed using the custom-made analysis program Maplab (MATLAB R2016a; MathWorks) (Potse et al., 2002). For all timepoints, AT and RT of all 224 electrodes were averaged from five consecutive beats. AT and RT were similarly determined as described for the catheter-derived EGM signals. SDR was calculated as the average and maximal difference in RT between two adjacent electrodes in the horizontal, vertical, transmural, and cubic orientation (Dunnink et al., 2017). The latter was the maximal difference in RT within four squared needles (Dunnink et al., 2017). Repolarization restitution curves were created using the ARI (RT–AT) of the needle electrodes. Averaged LV and RV recordings were used to assess global ventricular RTs. Diastolic interval was calculated as cycle length –QT.

### Arrhythmia Quantification

Animals were defined to be inducible when three or more episodes of TdP occurred during the 10-min period after the onset of dofetilide administration. TdP were defined as a polymorphic ventricular tachycardia of five beats or more, twisting around the isoelectric line.

During this 10-min interval, an arrhythmia score (AS) was calculated to quantify the severity of the arrhythmic activity in the experiments. Ectopic events were scored according to the *n* + 1 rule, wherein *n* reflects the number of ectopic beats. Persistent TdP were scored according to the number of defibrillation shocks needed to terminate the TdP; one defibrillation was awarded 50 points, two cardioversions were given 75 points, and three or more shocks were given 100 points. AS was then calculated by averaging the scores of the three most severe arrhythmic episodes within the 10-min interval (Stams et al., 2016).

### Statistical Analysis

All obtained data are expressed as mean ± standard deviation (SD). Statistical analyses and comparison of serial electrophysiological data were performed with (un)paired Student *t*-tests. Inducibility was analyzed using the McNemar test. AS was analyzed with the Wilcoxon signed rank test, and the Mann–Whitney *U* test was used for the number of TdP. *P* < 0.05 was considered statistically significant.

## Results

### Dofetilide Experiments

In the IVR experiments, a trend toward further prolongation in cycle length was observed following administration of dofetilide (RR from 1,403 ± 271 to 1,569 ± 392 ms, *p* = 0.07), but this was not accompanied by changes in QRS morphology. In comparison to the IVR experiments, pacing from the RVA (1,000 ± 0 ms) significantly delayed LV activation (53 ± 8 vs. 20 ± 6 ms in IVR experiments, *p <* 0.05) and shortened LV MAPD/ARI and RV MAPD from 313 ± 45 and 251 ± 29 ms to 254 ± 28 and 228 ± 25 ms, respectively (both *p <* 0.05 vs. IVR experiments). The differences in pacing rate did not affect temporal dispersion of repolarization, as baseline LV and RV STV did not differ between both rhythm strategies (Table 1).

**TABLE 1:** Serial comparison of the electrophysiological effects of IVR and acute RVA60 conditions in baseline and following dofetilide administration (both groups *n* = 8).

	**IVR**			**RVA60**		
**Parameters (ms)**	**Baseline**	**Dofetilide**	**% Increase**	**Baseline**	**Dofetilide**	**% Increase**
PP	551 ± 75	748 ± 133**	36	573 ± 101	874 ± 187***	53
RR	1,403 ± 271	1,569 ± 392	12	1,000 ± 0^+^	1,000 ± 0^+^	0
QRS	95 ± 12	97 ± 11	2	115 ± 9^+^	116 ± 7^+^	0
QT	374 ± 51	549 ± 145**	47	384 ± 44	563 ± 76***	47
QTc	339 ± 50	500 ± 119**	47	384 ± 44	563 ± 76***	47
JT	244 ± 48	403 ± 112**	65	268 ± 44	448 ± 76***	67
JTp	195 ± 26	267 ± 58*	40	159 ± 25^+^	231 ± 42***	46
Tp-e	84 ± 44	185 ± 90*	151	108 ± 33	215 ± 61***	108
LV-AT	20 ± 6	21 ± 5		53 ± 8^+^	56 ± 9^+^	
RV-AT	28 ± 7	29 ± 8		29 ± 7	31 ± 10	
ΔAT	−11 ± 5	−9 ± 4		23 ± 10^+^	25 ± 17^+^	
LVMAPD/LVARI	313 ± 45	462 ± 108*	48	254 ± 28^+^	417 ± 58***	64
RVMAPD	251 ± 29	335 ± 78*	33	228 ± 25^+^	330 ± 47***	45
ΔMAPD	40 ± 33	112 ± 53*	180	27 ± 18	107 ± 48**	296
LV-RT	332 ± 50	484 ± 107*	46	307 ± 30	473 ± 58***	54
RV-RT	280 ± 34	365 ± 80*	30	257 ± 27	361 ± 53**	40
ΔRT	30 ± 38	102 ± 55*	240	50 ± 17	131 ± 58*	162
STV_LV	2.0 ± 1.1	4.3 ± 1.6***	115	1.3 ± 1.4	2.8 ± 2.2*	115
STV_RV	1.0 ± 1.1	2.0 ± 1.1*	100	1.3 ± 1.5	2.4 ± 2.4	85
Inducibility, %	1 ± 0	75		1 ± 0	13^+^	
AS	0.0 ± 0.0	37 ± 27		0.0 ± 0.0	8 ± 12^+^	
Average n TdP		9.5 ± 12.3			0.8 ± 1.8^+^	

Values are represented as mean ± SD.

*p < 0.05, **p < 0.01, ***p < 0.001 vs. baseline.

*Comparison IVR experiment - RVA60 experiment:*

*^+^*p <* 0.05 vs. IVR experiment.*

*AS, arrhythmia score; ΔAT, interventricular differences in activation time (calculated as ΔAT = LV-AT – RV-AT); ΔAPD, interventricular dispersion of repolarization (calculated as ΔAPD = LV MAPD/ARI – RV MAPD); ΔRT, interventricular dispersion of repolarization time (calculated as ΔRT = LV-RT – RVRT); LV-AT, left ventricular activation time; LVARI, left ventricular activation recovery interval; LVMAPD, left ventricular monophasic action potential duration (measured at 80% repolarization); LV-RT, left ventricular repolarization time (calculated as LV-RT = LV-AT + LVMAPD/ARI); RV-AT, right ventricular activation time; RVMAPD, right ventricular monophasic action potential duration (measured at 80% repolarization); RV-RT, right ventricular repolarization time (calculated as RV-RT = RV-AT + RVMAPD); STV_LV, short-term variability of repolarization (from 30 consecutive LV MAPD/ARI); STV_RV, short-term variability of repolarization (from 30 consecutive RV MAPD).*

Dofetilide administration caused a similar prolongation of the QTc from 330 ± 50 and 384 ± 44 ms to 500 ± 119 and 563 ± 76 ms in IVR and RVA60 experiments, respectively (both *p <* 0.05). Moreover, dofetilide caused a substantial prolongation of LV MAPD/ARI, RV MAPD, and ΔMAPD under both rhythm strategies (Table 1). Even though no statistically significant differences herein were observed between IVR and RVA60 conditions, these dofetilide-induced prolongations did seem to be more pronounced in RVA60 experiments, as these values depicted a greater relative increase in duration (Figure 1B and Table 1). Nevertheless, absolute LV and RV STV values following dofetilide infusion, as well as their relative increases in comparison to baseline, did not differ between the two different experimental settings.

**FIGURE 2:**
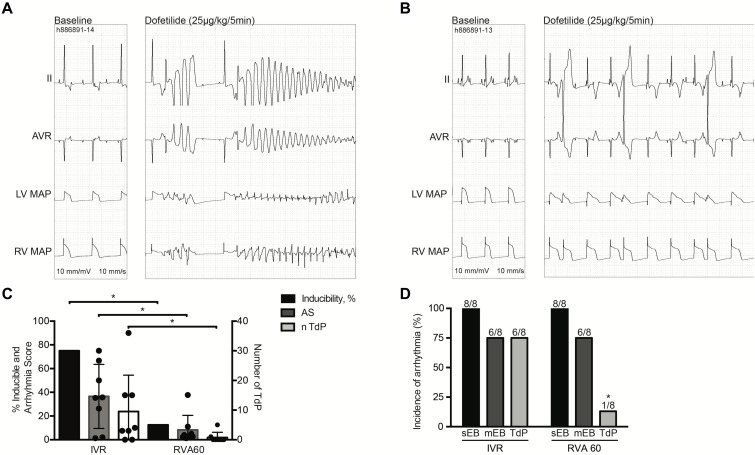
Dofetilide experiments performed under IVR or RVA60 conditions in chronic atrioventricular block dogs. Representative ECG traces (lead II and AVR) associated with MAPs recorded from the LV and RV at baseline and after dofetilide (25 μg/kg per 5 min) infusion in serial IVR **(A)** and RVA60 **(B)** experiments. Quantification of TdP-inducible CAVB dogs, AS, and average number of TdP. The circular dots represent the different experiments **(C)**. The incidence of sEB, mEB, and TdP under IVR and RVA60 conditions **(D)**. Values are represented as mean ± SD. AS, arrhythmia score; CAVB, chronic atrioventricular block; ECG, electrocardiogram; IVR, idioventricular rhythm; LV/RV MAP, left/right ventricular monophasic action potential; mEB, multiple ectopic beat; RVA60, right ventricular apex paced at 60 beats per minute; sEB, single ectopic beat; TdP, torsade de pointes arrhythmia. *p < 0.05 vs. IVR.

Nevertheless, TdP inducibility of 75% (6/8) in IVR experiments was significantly higher than the 13% (1/8) observed in the RVA60 experiments (*p* < 0.05) (Table 1 and Figures 2A–C). Moreover, the IVR experiments displayed more severe arrhythmic events (AS: 37 ± 27 vs. 8 ± 12 in RVA60 experiments, *p <* 0.05; Table 1 and Figures 2C,D). Collectively, these results demonstrated and confirmed that the slowing of heart rate increased the incidence and severity of arrhythmic events in the CAVB dog.

### Mapping Experiments

Detailed mapping experiments (*n* = 10) established the effects of stepwise lowering heart rate on spatial heterogeneity in repolarization. Lowering pacing rate from high to middle rate pacing prolonged repolarization as demonstrated by QTc and LV ARI from 344 ± 12 and 202 ± 15 to 365 ± 15 and 229 ± 17, respectively, both *p <* 0.05 (Table 2). This effect was also reflected in the RT of the local EGMs (LV RT from 268 ± 19 to 295 ± 19 ms; RV RT from 244 ± 19 to 257 ± 14 ms; both *p <* 0.05). Subsequent lowering to experimental rate caused a further prolongation of repolarization; LV ARI to 260 ± 19 ms (*p* < 0.05 vs. high and middle rate) and RT to 325 ± 20 ms (LV) and 279 ± 15 ms (RV) (both *p <* 0.05 vs. high and middle rate). Moreover, although insignificant, a modest trend toward increasing temporal dispersion seemed to appear as rate was decreased from high to middle rate (from 0.9 ± 0.4 to 1.2 ± 0.5 ms, *p* = 0.31).

**TABLE 2:** Serial comparison of the electrophysiological effects of high, middle, and experimental rate pacing (*n* = 10).

**Parameters (ms)**	**High rate 128 ± 10 bpm**	**Middle rate 88 ± 10 bpm**	**Experimental rate 61 ± 3 bpm**
PP	582 ± 63	565 ± 67	552 ± 65
RR	471 ± 46	690 ± 77*	986 ± 45^*+^
QRS	112 ± 6	111 ± 7	109 ± 8
QT	298 ± 12	338 ± 14*	368 ± 28^*+^
QTc	344 ± 12	365 ± 15*	369 ± 27*
JT	186 ± 13	226 ± 17*	259 ± 31^*+^
JTp	128 ± 19	159.23*	184 ± 37^*+^
Tp-e	58 ± 11	68 ± 12*	75 ± 19^*+^
LVMAPD/LVARI	202 ± 15	229 ± 17*	260 ± 19^*+^
STV_LV	0.9 ± 0.4	1.2 ± 0.5	1.4 ± 0.8
RV-AT	55 ± 15	51 ± 10	49 ± 9
LV-AT	65 ± 13	67 ± 10	66 ± 9
RV-RT	244 ± 19	257 ± 14*	279 ± 15^*+^
LV-RT	268 ± 19	295 ± 19*	325 ± 20^*+^
Transmural dispersion	14 ± 2	15 ± 2*	20 ± 4^*+^
Vertical dispersion	25 ± 4	28 ± 7	33 ± 7^*+^
Horizontal dispersion	24 ± 4	27 ± 5*	34 ± 8^*+^
Cubic dispersion	33 ± 5	36 ± 6*	46 ± 11^*+^
**Maximal dispersion**			
Transmural dispersion	45 ± 9	50 ± 10	62 ± 12^*+^
Vertical dispersion	53 ± 12	57 ± 14	71 ± 15^*+^
Horizontal dispersion	55 ± 14	60 ± 16	76 ± 24^*+^
Cubic dispersion	60 ± 14	69 ± 17	84 ± 22^*+^

Values are represented as mean ± SD.

*p < 0.05 vs. high rate pacing.

*^+^*p* < 0.05 vs. middle rate pacing.*

*LV-AT, left ventricular activation time; LVARI, left ventricular activation recovery interval; LVMAPD, left ventricular monophasic action potential duration (measured at 80% repolarization); LV-RT, left ventricular repolarization time (calculated as LV-RT = LV-AT + LVMAPD/ARI); RV-AT, right ventricular activation time; RVMAPD, right ventricular monophasic action potential duration (measured at 80% repolarization); RV-RT, right ventricular repolarization time (calculated as RV-RT = RV-AT + RVMAPD); STV_LV, short-term variability of repolarization (from 30 consecutive LV MAPD/ARI).*

**FIGURE 3:**
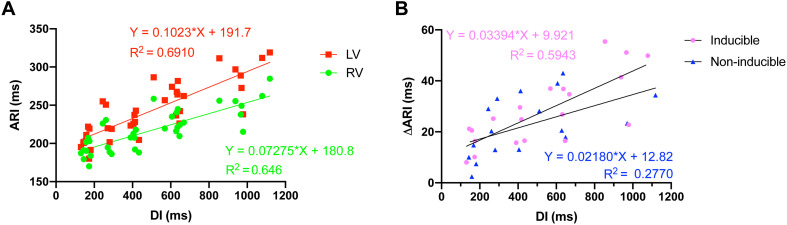
Action recovery interval (ARI) restitution curves. **(A)** Increasing cycle length, and thus diastolic interval (DI), caused interventricular dispersion of ARI as the repolarization duration of the left ventricle prolonged more than the right ventricle. **(B)** The interventricular dyssynchrony in ARI prolongation (DARI) was more pronounced in animals that were to become inducible (*n* = 5) when challenged with dofetilide, compared to noninducible animals (*n* = 5).

ARI restitution curves were dissimilar for the LV and the RV. The steeper slope of the LV curve reflected the greater prolongation of LV ARI in response to increasing cycle lengths (Figure 3A). A comparison of the animals that became inducible when challenged with dofetilide to the animals that were TdP resistant (non-inducible) showed that the interventricular difference in ARI prolongation was greater in the inducible animals (Figure 3B).

**FIGURE 4:**
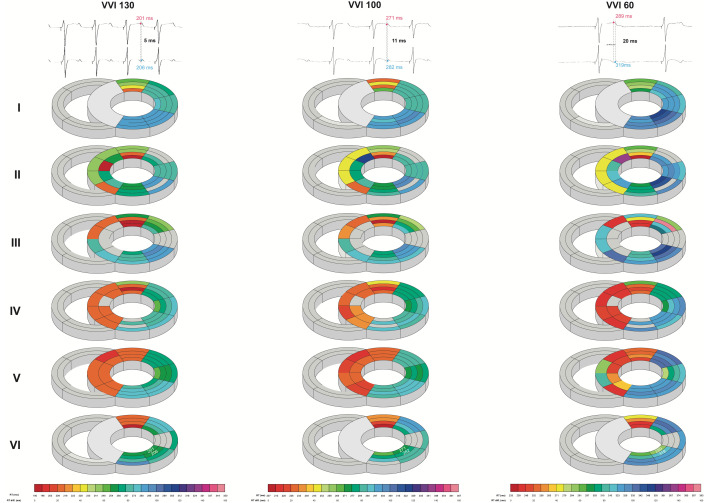
Bradycardia-induced increases in left ventricular intraventricular spatial dispersion of repolarization. Local repolarization times (RT) of one animal during high (130 beats/min), middle (100 beats/min), and experimental (60 beats/min) rate in the septum and LV from base (I) to apex (VI). Traces of two unipolar electrograms with their corresponding RT and their difference herein are depicted to illustrate the evolution of spatial dispersion. Colors and color gradients correspond to the absolute RT and ΔRT values, respectively, as depicted in the color bars below the cardiac maps.

Intraventricularly, SDR in the LV increased as pacing rate was stepwise decreased, starting from a rate similar to canine sinus rhythm. This increase was most prominent under the most bradycardic conditions, as average and maximal SDRs were significantly increased in all orientations compared to both high- and middle-rate pacing (Table 2 and Figure 4). This effect was most pronounced in the cubic orientation; decreasing pacing frequency from high to middle to experimental rate increased the maximal cubic dispersion from 60 ± 14 to 69 ± 17 ms to 84 ± 22 ms, respectively (*p* < 0.05 vs. high and middle rate).

## Discussion

This study established the contribution of severe bradycardia in arrhythmogenesis and correlated its proarrhythmic effects with changes in electrophysiological parameters. The results have confirmed that severe bradycardia increases (1) the likelihood of arrhythmia development and (2) the severity of arrhythmic events and show for the first time *in vivo* that this can be explained by (3) bradycardia-induced increases in SDR in three dimensions, and (4) to a much lesser extent temporal dispersion of repolarization.

### Bradycardia as a Modulator of Arrhythmogenesis in the CAVB Dog Model

The two main characteristics of the CAVB dog—the compensated heart failure combined with its increased susceptibility for TdP—which both result from ventricular remodeling, make this animal model unique in the research field of ventricular arrhythmias. Also in humans, cardiac remodeling that results in compensated heart failure is associated with an increased risk of SCD as the result of ventricular arrhythmias.

This disposition for ventricular arrhythmias arises from multiple electrical alterations that cumulatively augment the likelihood that ectopic stimuli are generated and enhance spatial differences that facilitate the spread and perpetuation of such stimuli. Hence, it creates a substrate, which facilitates the development of ectopic beats and TdP. However, an additional trigger is needed to provoke such events. These triggers initiate ectopic stimuli by destabilizing cellular electrophysiology to such extent that early or late after-depolarization is provoked; this increasing instability is reflected by the temporal dispersion of repolarization, quantified as the STV (Thomsen et al., 2004). In most experiments, a gradual progression from single to multiple ectopic beats to TdP can be observed in the course of the experiment. This evolution of arrhythmic events has been demonstrated to mainly rely on increases in SDR (Dunnink et al., 2017). As such, in comparison to the first ectopic beat, STV is not further increased prior to the first TdP, whereas the SDR has become significantly higher (Smoczynska et al., 2019; van Weperen et al., 2019).

In the serial comparison, dofetilide induced ectopic events in all animals, regardless of rhythm strategy. Nevertheless, in RVA60-paced animals, these events were limited to single and multiple ectopic beats, whereas TdP were abundantly present in the IVR tested animals (Figure 2). This difference in severity of arrhythmic events was also reflected in the significantly higher AS in IVR experiments (Table 1).

However, the increased incidence and severity of arrhythmic events in IVR experiments were not reflected in the dofetilide-induced increases in repolarization duration and/or STV (Figure 1 and Table 1). This discrepancy complies with the aforementioned paradigm on arrhythmogenesis in the CAVB dog model, whereby temporal dispersion of repolarization is involved in the initiation of arrhythmic events, and SDR becomes of increasing importance in the perpetuation of progression of arrhythmic events. Hence, the results obtained in the serial comparison suggest that developing bradycardia encourages arrhythmogenesis through increasing SDR, which results in more severe TdP. Interestingly, although insignificantly different, dofetilide appeared to induce a greater increase in Tp-e in the IVR than in the RVA experiments.

Correspondingly, the additional mapping experiments that studied SDR under baseline conditions demonstrated that heterogeneities in interventricular and intraventricular repolarization durations increased as pacing frequency was gradually lowered. Interventricular dispersion of repolarization is known to be of importance in TdP arrhythmogenesis and to be a bradycardia-dependent phenomenon (Verduyn et al., 1997; Meijborg et al., 2015). As ΔAPD reflects the dispersion of repolarization over a larger area, this parameter of heterogeneity might be less informative on arrhythmogenic consequences than intraventricular SDR, as the arrhythmogenic potential mainly relies on the steepness of (local) repolarization gradients. Nevertheless, as we see a clear rate dependency of interventricular dispersion (Figure 3), this clearly indicates that the global (in)stability of repolarization is profoundly affected by changes in heart rate. In addition, the aforementioned local repolarization gradients were assessed in the mapping experiments. Dunnink et al. (2017) have demonstrated the importance of this intraventricular SDR in arrhythmogenesis, as they showed that dofetilide-induced TdP are associated with increases in intraventricular SDR and that dispersion becomes higher in inducible animals than noninducible animals (Dunnink et al., 2017). Furthermore, our results of the serial comparison show great similarities to a study by Bossu et al. (2018). In their study, infusion of the antiarrhythmic drug GS-458967 caused a reversal of dofetilide-induced increases in intraventricular SDR, which was associated with the complete abolishment of TdP, but not of single and multiple ectopic beats (Bossu et al., 2018). Hence, severe bradycardia appears to cause heterogeneous electrical alterations that result in an increased incidence and severity of arrhythmic events. This effect is mainly reflected in an increased interventricular and intraventricular SDR and to a lesser extent the temporal dispersion of repolarization.

These results correspond to prior studies reporting an increased SDR under bradycardic conditions. For example, Kim et al. (2013), showed that bradycardia altered calcium handling in their Langendorff-perfused rabbit hearts, and that this alteration augmented SDR, thereby facilitating ventricular ectopy. Moreover, multiple studies have identified bradycardia as a modulator of ventricular arrhythmogenesis in both acquired and congenital long-QT syndromes. Shimizu and Antzelevitch (1998) demonstrated how bradycardia-induced prolongation of APD increased the transmural dispersion of repolarization in a perfused RV-wedge model, facilitating the development of TdP. Similarly, Restivo et al. (2004) investigated the effects of bradycardia on SDR *in vivo* in a guinea pig model of long-QT syndrome three. They demonstrated that bradycardia augmented the baseline heterogeneities in APD, which promoted ventricular arrhythmogenesis. However, in contrast to the current study, these studies were limited in their assessment of repolarization to either a small number of RV sites or the epicardium, precluding the evaluation of global ventricular changes. Hence, while prior studies have underlined the importance of bradycardia as a modulator of arrhythmogenesis and demonstrated its effects on SDR, the current study is unique in its high-resolution, global, three-dimensional evaluation of these effects in an *in vivo* large animal model.

Nevertheless, even though the aforementioned studies and the current study demonstrate a clear correlation between heart rate and changes in dispersion of repolarization, instability of other electrical properties, for example, myocardial activation, might have also played into the observed episodes of arrhythmogenesis.

Interestingly, dofetilide tended to prolong the PP intervals more in the RVA experiments than in the IVR experiments. Hence, a heightened sympathetic tone in the more bradycardic IVR experiments could have promoted the more arrhythmogenic outcome of these experiments.

### Clinical and Experimental Implications

For the experimental setting, these results emphasize the importance of carefully considering rhythm control in studies in the field of arrhythmogenesis and antiarrhythmic interventions. For example, in studies testing drugs, it is important that one is able to discriminate between arrhythmogenic effects related to the drug tested and experimental intrinsic effects, such as the administration of anesthetics (Dunnink et al., 2010). The identification of acute bradycardia as an additional experimental design–related proarrhythmic factor thus encompasses multiple implications for future experiments, one of which being that the presence of severe bradycardia might lead to an overestimation or underestimation of the proarrhythmic or antiarrhythmic efficacy of drugs and/or therapies, respectively.

In the clinical setting, it is important to understand how heart rate directly affects cardiac electrophysiology and how it can both contribute to and impede ventricular arrhythmogenesis. Especially the latter observation is of great clinical interest. The observation that arrhythmogenesis can be impeded by increasing pacing rate highlights the potential of pacemakers to treat chronic cardiac conditions wherein there is an increased risk of ventricular arrhythmogenesis.

### Limitations

Inconsistent ventricular activation is known to cause electrophysiological instability, possibly evoking an electrical storm. In IVR experiments, the uncontrollability of ventricular activation focus might have been an additional proarrhythmic factor. However, no changes in QRS morphologies were observed in the serial comparisons, implying that instability of ventricular activation is unlikely to have caused the increased arrhythmic activity observed in the IVR experiments.

In addition, as dofetilide infusion is stopped prematurely when TdP occur within the 5-min window of infusion, less dofetilide was administered in IVR experiments. Therefore, the obtained result might still be an underestimation of the proarrhythmic effects of severe bradycardia.

Moreover, pacing the animals in the serial experiments could have been a confounding factor as eliciting an activation front may have interfered with arrhythmogenesis. Nevertheless, as we see a similar increase in STV and observe a comparable initiation of ectopic events, we believe that the increased TdP incidence in IVR experiments is a result of the greater increase in SDR. As we show that the extent of SDR closely correlates to heart rate, we do not believe that cardiac pacing was responsible for the difference in arrhythmogenesis.

Lastly, the frequency–dependency of SDR was only studied under baseline conditions and not additionally tested following the administration of dofetilide. Hence, these mapping experiments demonstrate that bradycardia induces proarrhythmic increases in SDR but do not directly demonstrate its role in arrhythmogenesis.

## Conclusion

Severe bradycardia in the CAVB dog increases the probability of arrhythmia development and the severity of arrhythmic events by heterogeneously causing cellular electrophysiological instability, which is mainly reflected in an increased spatial, and to a lesser extent temporal, dispersion of repolarization.

## Data Availability Statement

The raw data supporting the conclusions of this article will be made available by the authors, without undue reservation.

## Ethics Statement

The animal study was reviewed and approved by The Committee for Experiments on Animals of Utrecht University, Netherlands.

## Author Contributions

VW, AD, AB, RV, VM, and JBe carried out the experiments. VW wrote the manuscript with support from MH and MV. JBa and RC helped supervise the project and revise the manuscript. MV supervised the project. All authors contributed to the article and approved the submitted version.

## Conflict of Interest

The authors declare that the research was conducted in the absence of any commercial or financial relationships that could be construed as a potential conflict of interest.
